# Treatment response, survival, safety, and predictive factors to chimeric antigen receptor T cell therapy in Chinese relapsed or refractory B cell acute lymphoblast leukemia patients

**DOI:** 10.1038/s41419-020-2388-1

**Published:** 2020-03-30

**Authors:** Limin Li, Jie Liu, Mengyuan Xu, Hongjuan Yu, Chengfang Lv, Fenglin Cao, Zhenkun Wang, Yueyue Fu, Mingwen Zhang, Hongbin Meng, Xiaoqian Zhang, Liqing Kang, Zhuo Zhang, Jinmei Li, Jiawei Feng, Xin Lian, Lei Yu, Jin Zhou

**Affiliations:** 1 Department of HematologyThe First Affiliated Hospital of Harbin Medical University, The Institute of The Hematology and Oncology of Heilongjiang Province, 150001 Harbin, China; 2 Shanghai Unicar-Therapy Bio-medicine Technology Co., Ltd, 201612 Shanghai, China

**Keywords:** Acute lymphocytic leukaemia, Acute lymphocytic leukaemia

## Abstract

This study aimed to evaluate treatment response, survival, safety profiles, and predictive factors to chimeric antigen receptor T cell (CAR-T) therapy in Chinese patients with relapsed or refractory B cell acute lymphoblast leukemia (R/R B-ALL). 39R/R B-ALL patients who underwent CAR-T therapy were included. Baseline data were collected from patients’ electronic medical records. Patients’ peripheral bloods, bone marrow aspirates, and biopsies were obtained for routine examination, and treatment response and survival profiles as well as adverse events were evaluated. The rates of complete remission (CR), CR with minimal residual disease (MRD) negative/positive, and bridging to hematopoietic stem-cell transplantation (HSCT) were 92.3%, 76.9%, 15.4%, and 43.6%, respectively. The median event-free survival (EFS) was 11.6 months (95% confidence interval (CI): 4.0–19.2 months) and median overall survival (OS) was 14.0 months (95% CI: 10.9–17.1 months). Bridging to HSCT independently predicted better EFS and OS, while high bone marrow blasts level independently predicted worse EFS. The incidence of cytokine release syndrome (CRS) was 97.4%, and refractory disease as well as decreased white blood cell independently predicted higher risk of severe CRS. Other common adverse events included hematologic toxicities (grade I: 5.1%, grade II: 7.7%, grade III: 17.9%, grade IV: 69.2%), neurotoxicity (28.2%), infection (38.5%), and admission for intensive care unit (10.3%). In conclusion, CAR-T therapy presents with promising treatment response, survival and safety profiles, and higher disease burden predicts worse survival as well as increased risk of severe CRS in Chinese R/R B-ALL patients.

## Introduction

Acute lymphoblastic leukemia (ALL), a heterogeneous hematological malignancy commonly arising from B cell precursor linage (B-ALL) and less commonly from T cell precursor linage (T-ALL), occurs in all ages but most prevalent in children with the incidence peaks at age from 3 to 5 years^1,2^. Owing to advances in understanding about pathogenesis of the disease, the development of novel targeted agents, optimized dosage, and schedule of combination chemotherapies as well as allogenic hematopoietic stem-cell transplantation (HSCT), the cure rates and survival length of B-ALL have been dramatically improved in the past decades^3^. However, despite the progress in cure rate and survival, relapse or refractory B-ALL (R/R B-ALL) is still an obstacle that worsens prognosis of both pediatric and adult B-ALL patients, which is also hard to be resolved by conventional salvage chemotherapy (complete remission (CR) lower than 30% and median OS of 4 months), or even HSCT^4–6^. Thus, new treatment strategies such as adoptive cellular therapies and antibody-based immune therapies are under investigation in order to rain benefits on management of R/R B-ALL.

Chimeric antigen receptor T cell (CAR-T) therapy is an adoptive cellular therapy that has achieved remarkable advances beyond conventional chemotherapies^7^. It genetically engineers patient-originated T cells to express an extracellular antigen-binding domain that binds cancer-specific antigens independent of major histocompatibility complex (MHC) and intracellular T cell signaling domain that leads to T cell activation and destruction of malignant cells^1^. The application of CAR-T has been implicated in acute leukemia and non-Hodgkin’s lymphoma, and it has made prominent progress in B-ALL during the past several years. Clinical studies in the United States and Europe illustrate that the CR rate to CAR-T therapy is around 90% in R/R B-ALL patients, which is similar to that in de novo B-ALL patients^8–14^. The survival profiles of R/R B-ALL patients have also been improved by CAR-T therapy, but with inevitable adverse events (such as cytokine release syndrome (CRS), B cell aplasia, neurotoxicity, and tumor lysis syndrome)^15,16^. As in China, the research on CAR-T therapy is still in its infancy and only one clinical trial investigates the treatment response and safety of CAR-T therapy for R/R B-ALL^17^. However, the study does not investigate the survival profiles or factors influencing the clinical outcomes by CAR-T therapy, leaving them still obscure in Chinese R/R B-ALL patients.

China is a populous country possessing a remarkably large number of B-ALL cases, including R/R B-ALL; however, the current treatment response to conventional therapies is still unsatisfactory and the donor resources of HSCT are limited, which leads to poor prognosis of B-ALL patients, especially in R/R B-ALL patients who have even worse response and survival profiles, and this poses a huge burden on medical and social resources^18,19^. It is known that CAR-T therapy is shown to greatly improve treatment response and survival of R/R B-ALL patients in the United States and Europe, but the relative investigations are still rare in China; therefore, active investigation of treatment response and survival benefits as well as predictive factors by CAR-T in Chinese R/R B-ALL patients is of great clinical value for future R/R B-ALL treatment in China.

In the current study, we aimed to evaluate the treatment response, survival, and safety profiles as well as prognostic factors to CAR-T therapy in Chinese R/R B-ALL patients.

## Materials and methods

### Patients

From August 2015 to May 2018, 39R/R B-ALL patients who underwent CAR-T cell therapy in The First Affiliated Hospital of Harbin Medical University were retrospectively included in this study. The inclusion criteria were: (1) diagnosed as CD19^+^ B-ALL by flow cytometry, bone marrow histology, and/or cytogenetics examinations; (2) presented with refractory disease or relapsed disease, which were defined as follows: refractory disease: failure to achieve CR at the end of induction therapy or complicated with extramedullary disease or sustained positive status of minimal residual disease (MRD) for 3 to 6 months; relapsed disease: reappearance of blasts in the blood or bone marrow (>5%) or in any extramedullary site after a CR (according to the National Comprehensive Cancer Network Guidelines of Acute Lymphoblastic Leukemia (Version 1.2018)); (3) underwent CAR-T cell therapy; (4) medical and follow-up records were complete and accessible. Patients were excluded if they were diagnosed as lymphoma or complicated with other malignancies. The present study was approved by the Institutional Review Boards of the The First Affiliated Hospital of Harbin Medical University, and the written informed consents were obtained from patients or their guardians.

### Baseline data collection

Baseline data were collected from patients’ electronic medical records, which included age, gender, previous chemotherapies (including induction, maintenance, and consolidation therapy), refractory disease, relapsed disease, disease burden before infusion (bone marrow blasts, extramedullary disease), fusion gene, gene mutation, white blood cell (WBC), lymphodepletion regimens, and characteristics of CAR-T cells.

### CAR-T cell production

After the eligibility of CAR-T cell therapy and the feasibility of manufacture of CAR-T cells were confirmed, patients’ peripheral blood mononuclear cells (PBMCs) were separated from leukapheresis products using lymphocyte separation medium. Then, CD3^+^ T cells were isolated from PBMCs using CD3 MicroBeads (Miltenyi Biotec GmbH, Shanghai, China), stimulated with anti-CD3/CD28 monoclonal antibodies (mAbs) 24 h for activation of T cells, and expanded using recombinant human interleukin-2 (IL-2) at 100 IU/mL for 48 h. The activated T cells were then transduced with supernatants of lentiviral vector, which could encode a chimeric T cell antigen receptor (the lentiviral vector consisted of a CD19/CD22-specific single-chain fragment variable (scFv), the transmembrane and cytoplasmic signaling domain of 4-1BB and the cytoplasmic signaling domain of CD3 ζ). The transduction was performed at multiplicity of infection 1:5–20 ratio in plates pre-coated with retronectin (Takara Biomedicals, Kusatsu, Japan). After transduction, transduced cells were cultured in complete medium X-VIVO 15 with 100 IU/mL IL-2 and 5 ng/mL IL-7 and IL-15, and expanded for 12 to 14 days. Finally, CAR-T cells were harvested, and the quality tests of T cells were carried out, which included transduction efficiency, purity, cell viability, CD4^+^ cell ratio, CD8^+^ cell ratio, killing ability in vitro, cytokine release capacity, quantity of endotoxin, mycoplasma, bacteria, fungus, and so on. Only all the quality tests met the quality control criteria can CAR-T cells be released for infusion into patients.

### Preparative lymphodepletion chemotherapy

Before CAR-T cell infusion, lymphodepletion was performed for the reduction of endogenous lymphocytes as well as tumor burden. According to prior therapies and prior adverse reactions to chemotherapy, 12 patients received cyclophosphamide (Cy) (300 mg/m^2^ for 5 days) plus fludarabine (Flu) (30 mg/m^2^) regimen and 27 patients were treated with other Cy-based (300 mg/m^2^) lymphodepletion regimens. After lymphodepletion chemotherapy, tumor burden of patients was re-assessed by imageology examination, physical examination, laboratory examination, MRD examination, and toxicity evaluation.

### CAR-T cell infusion

CAR-T cell infusion was conducted on day 2 or day 3 after the lymphodepletion chemotherapy. Pretreatments with acetaminophen and diphenhydramine were administered 30–60 min before CAR-T cell infusion to prevent infusion reactions related to cryopreservants, and the patients or their caregivers were instructed to report symptoms, such as shortness of breath, rash, chills, chest pain, and back pain. At the time of infusion, oxygen, suction, and emergency medications, including adrenaline, were readily available. Patients were infused with a dose ranging from 3×10^6^/kg to 10×10^6^/kg freeze-thawed CAR-T cell solution through the largest patent lumen without a filter and an infusion pump, and the infusion process was completed within 10–15 min based on the patients’ tolerance. Generally, a dose escalation infusion or a one-time infusion was performed. As for dose escalation infusion, the infused dose was 10%, 30%, and 60% of the expected total dose according to infusion reactions, respectively. The infusion of CAR-T cells would be stopped if a severe infusion-related reaction occurred, and symptomatic treatment would be administered as necessary. The infusion could be resumed at a 50% rate reduction after resolution of symptoms. Vital signs were monitored closely during the infusion, and the monitoring of blood-oxygen saturation was carried out every 15 min before, during, and after infusion until the patient was in stable condition. Blood samples were collected at 20 min and 2 h after infusion, and the levels of CAR-T cells, blood potassium, and uric acid were detected.

### Post-infusion monitoring management

Post-infusion monitoring and management were administered to patients after CAR-T cell infusion, and the monitoring included daily history and physical examination, blood routine, blood product transfusion, monitoring for disseminated intravascular coagulation, monitoring for tumor lysis syndrome, profiling of serum levels of liver enzymes, albumin and fractionated bilirubin, and infectious disease as well as serum C-reactive protein, cytokines, and ferritin monitoring for CRS and hemophagocytic lymphohistiocytosis, and so on. Further, symptomatic treatments of CRS were carried out as well. CRS grading was performed at least twice a day when a change in the patient’s clinical status occurred. For patients who developed severe CRS, they were transferred into intensive care unit (ICU), and anti-IL-6 therapy with tocilizumab and administration of corticosteroids were performed according to the clinical symptoms.

### Sample collection

Peripheral bloods were extracted daily for at least 7–10 days and then weekly for 4 weeks after CAR-T cell infusion for complete differential blood counts, comprehensive chemistry profiles, flow cytometry, and quantitative polymerase chain reaction (qPCR) analysis. Bone marrow puncture was performed every 3–5 days for 4 weeks following CAR-T cell infusion and then as clinically indicated, which conducted for morphologic examination, measurement of vector copy number of CAR-T cells (also detected in peripheral bloods) using quantitative real time-PCR, and assessments of remission as well as MRD. Further bone marrow analyses were determined by the treating physician. Besides, lumbar puncture and exfoliative cytology examination of cerebrospinal fluid were also carried out 1 week after CAR-T cell infusion.

### Subsequent therapy

After CAR-T cell infusion, 36 patients achieved CR, and 3 patients failed to remission. Among 36 patients with CR, 17 patients were confirmed eligible for HSCT by treating physician and bridged to HSCT within 2 months following CAR-T cell infusion. All 17 HSCT patients did not undergo maintenance treatment, and there was no one suffering from relapse after HSCT. As for the other 19 CR patients, 8 cases repeated CAR-T cell infusion for consolidation therapy; 7 cases received no further therapy until they developed relapsed disease; and 4 cases underwent consolidation chemotherapy. Among 3 patients without CR, two patients were present with chemoresistance and did not receive further therapy (died subsequently), and one patient received salvage chemotherapy and still survived.

### Follow-up

Patients would be discharged if stable for 72 h and there was no evidence of significant tumor lysis, transfusion reactions, or unforeseen adverse reactions after CAR-T cells infusion. (Generally, patients with CRS grade 1–2 were hospitalized for 5 to 7 days, and 2 to 3 weeks for patients with CRS grade 3–4.) After hospital discharge, patients were followed up weekly for 4 weeks, monthly for 6 months, and quarterly for 3 years (intensive follow-up was performed as clinically indicated). Last follow-up date was 30 July 2018, and the median follow-up duration was 9.5 months (range: 0.9–34.3 months). During the follow-up period, patients were monitored for disease status, and the physical examination, document of toxicity and laboratory examinations of blood samples, and survival status of CAR-T cells were performed at each visit. Meanwhile, adverse events occurred after CAR-T cells infusion and HSCT were recorded as well, which included symptoms of CRS (fever, hypotension, hypoxemia, capillary leak syndrome, hepatic injury, heart injury), hematologic toxicities, neurotoxicity, infection, graft vs. host disease (GVHD), erythra, gastrointestinal reactions, hemorrhagic cystitis, and hepatic veno-occlusive disease. Grading of toxicity was referring to the National Cancer Institute Common Terminology Criteria for Adverse Events, version 5.0^20^, and the GVHD was assessed according to the National Institutes of Health (NIH) Consensus Working Group criteria.

### Definitions

CR was defined as <5% bone marrow blasts, the absence of circulating blasts, and no extramedullary disease, regardless of cell count recovery^9^. MRD-negative status was defined as the presence of leukemic cells in the bone marrow was <0.01% by means of flow cytometry or the fusion genes (such as *BCR/ABL1*, *E2A/PBX1*, *MLL*, *TEL/AML1*) detected by qPCR turned negative. Treatment failure was defined as no remission after CAR-T cell infusion. Relapse was defined as the reappearance of blasts in the blood or bone marrow (>5%) or in any extramedullary site after a CR. Event-free survival (EFS) was defined as the interval from CAR-T cell infusion to treatment failure, relapse, death, or the final follow‑up. Overall survival (OS) was defined as the interval from CAR-T cell infusion to death or the final follow‑up. CRS was graded according to the guideline^21^, and severe CRS was defined as grade 3 or higher.

### Statistical analysis

SPSS 22.0 statistical software (SPSS Inc., Chicago, IL, USA) was used for statistical data processing, and the GraphPad Prism 7.00 software (GraphPad Software, La Jolla, CA, USA) was used for creating figures. Count data were expressed as count (percentage), and the comparison was determined by the *χ*^2^ test; continuous data were described as mean value ± standard deviation if normally distributed and as median (range) if not normally distributed. Kaplan–Meier curve was used to demonstrate survival profiles, and the log-rank test was used to determine the survival difference between subgroups. Univariate and multivariate Cox’s proportional hazards regression model analyses were performed to evaluate the prognostic factors, and the multivariate Cox’s proportional hazards regression model analysis was performed using Forward Stepwise (Conditional likelihood ratio) method. Reported statistical significance levels were all two-sided. *P* value <0.05 was considered significant.

## Results

### Patients’ characteristics

The median age for 39R/R B-ALL patients was 13 (range 1–57) years, and the number of patients aged <18, 18–30, and 31–60 years were 27 (69.2%), 5 (12.8%), and 7 (18.0%), respectively (Table 1). There were 36 (92.3%) patients with refractory disease and 17 (43.6%) patients with relapsed disease. In addition, 9 (23.1%) patients had bone marrow blasts ≥5% and 22 (56.4%) patients were with extramedullary disease. There were 12 (30.8%) patients received Flu + Cy lymphodepletion regimen and 27 (69.2%) received non-Flu + Cy regimen. Other detailed characteristics were listed in Table 1.

**Table 1:** Characteristics of patients with R/R B-ALL.

Characteristics	R/R B-ALL patients (*N* = 39)
Age (years)	13 (1–57)
<18	27 (69.2)
18–30	5 (12.8)
31–60	7 (18.0)
Gender (male/female)	22/17
Number of previous chemotherapies	4 (2–15)
2–4	22 (56.4)
5–10	15 (38.5)
>10	2 (5.1)
Refractory disease (*n*/%)	36 (92.3)
Relapsed disease (*n*/%)	17 (43.6)
Bone marrow blasts ≥5%	9 (23.1)
Extramedullary disease	22 (56.4)
CNSL	12 (30.7)
Fusion gene (*n*/%)	
*BCR*/*ABL1*	12 (30.8)
Others^a^	8 (20.5)
Gene mutation	
SH2B3	15 (38.5)
PAX5	13 (33.3)
Others^b^	23 (69.0)
WBC (×10^9^/L)	35.76 (0.70–850.60)
Lymphodepletion regimens	
Flu + Cy	12 (30.8)
Non-Flu + Cy	27 (69.2)
CAR-T cells	
Anti-CD19	25 (64.1)
Anti-CD19 + CD22	14 (35.9)

Data were presented as median (range) or count (percentage).

*B-ALL* B cell acute lymphoblastic leukemia, *CR* complete remission, *CNSL* central nervous system leukemia, *WBC* white blood cell, *Flu* fludarabine, *Cy* cyclophosphamide, *CAR-T* chimeric antigen receptor T cells.

^a^Others: E2A/PBX1, EVI1, MLL/AF4, TEL/AML1.

^b^Others: PAX5, TP53, IL7R, FLT3, IKZF1, NT5C2, NOTCH1, PTEN, TET2.

### Treatment response to CAR-T therapy

**Fig. 1:**
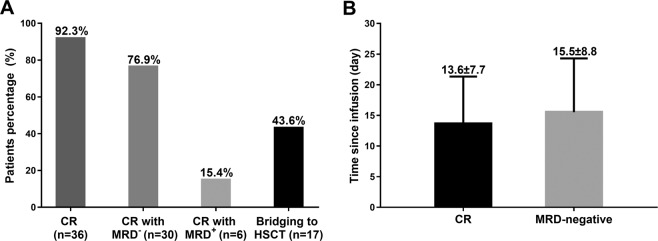
Treatment response to CAR-T therapy in R/R B-ALL patients. CR rate was 92.3%, CR with MRD-negative rate was 76.9%, CR with MRD-positive rate was 15.4%, and bridging to HSCT rate was 43.6% (**a**). The mean time since infusion to CR was 13.6 ± 7.7 days, and the mean time since infusion to MRD negative was 15.5 ± 8.8 days (**b**). CR, complete remission; MRD, minimal residual disease; HSCT, hematopoietic stem-cell transplantation.

The rates of CR, CR with MRD negative, CR with MRD positive, and bridging to HSCT were 92.3%, 76.9%, 15.4%, and 43.6%, respectively, for R/R B-ALL patients underwent CAR-T therapy (Fig. 1a). The mean time of progression to CR after infusion was 13.6 ± 7.7 days, and the mean time of progression to MRD negative after infusion was 15.5 ± 8.8 days (Fig. 1b).

### Factors affecting treatment response to CAR-T therapy

In order to investigate factors that possibly influence CR, patients were divided into subgroups depending on their characteristics (Table 2). We found that the CR rate was numerically higher in the subgroup with lower WBC concentration compared to that with higher WBC concentration (*P* = 0.079). None of the other characteristics was correlated with CR rate in R/R B-ALL patients who underwent CAR-T therapy (all *P* > 0.05). Moreover, both univariate and multivariate logistic regression analysis disclosed that none of the characteristics affected CR in R/R B-ALL patients (all *P* > 0.05) (Table 3). It was also interesting to note that CNSL (yes vs. no) did not influence CR (*P* = 0.920).

**Table 2:** Subgroup analysis of CR.

Characteristics	CR (n/%)	Non-CR (*n*/%)	*P* value
Age			0.229
<18 years	24 (88.9)	3 (11.1)	
≥18 years	12 (100.0)	0 (0.0)	
Gender			0.709
Female	16 (94.1)	1 (5.9)	
Male	20 (90.9)	2 (9.1)	
Number of previous chemotherapies			0.401
<4	15 (88.2)	2 (11.8)	
≥4	21 (95.5)	1 (4.5)	
Refractory disease			0.401
No	21 (95.5)	1 (4.5)	
Yes	15 (88.2)	2 (11.8)	
Relapsed disease			0.542
No	4 (100.0)	0 (0.0)	
Yes	32 (91.4)	3 (8.6)	
Bone marrow blasts			0.323
<5%	27 (90.0)	3 (10.0)	
≥5%	9 (100.0)	0 (0.0)	
Extramedullary disease			0.709
No	16 (94.1)	1 (5.9)	
Yes	20 (90.9)	2 (9.1)	
CNSL			0.920
No	25 (92.6)	2 (7.4)	
Yes	11 (91.7)	1 (8.3)	
BCR/ABL1			0.920
Negative	25 (92.6)	2 (7.4)	
Positive	11 (91.7)	1 (8.3)	
SH2B3 mutation			0.269
Negative	23 (95.8)	1 (4.2)	
Positive	13 (86.7)	2 (13.3)	
PAX5 mutation			0.202
Negative	25 (96.2)	1 (3.8)	
Positive	11 (84.6)	2 (15.4)	
WBC			0.079
<30 × 10^9^/L	19 (100.0)	0 (0.0)	
≥30 × 10^9^/L	17 (85.0)	3 (15.0)	
Lymphodepletion regimens			0.920
Flu + Cy	11 (91.7)	1 (8.3)	
Non-Flu + Cy	25 (92.6)	2 (7.4)	
CAR-T cells			0.248
Anti-CD19	24 (96.0)	1 (4.0)	
Anti-CD19 + CD22	12 (85.7)	2 (14.3)	

Data were presented as count. Comparison was determined by *χ*^2^ test. *P* value <0.05 was considered significant.

*CR* complete remission, *CNSL* central nervous system leukemia, *WBC* white blood cell, *Flu* fludarabine, *Cy* cyclophosphamide, *CAR-T* chimeric antigen receptor T cells.

**Table 3:** Factors affecting CR by logistic regression analysis.

Parameters	Logistic regression model			
	*P* value	OR	95% CI	
			Lower	Higher
Univariate logistic regression				
Age (≥18 vs. <18 years)	0.999	–	0.000	–
Gender (male vs. female)	0.711	0.625	0.052	7.530
Number of previous chemotherapies (≥4 vs. <4)	0.418	2.800	0.232	33.779
Refractory disease (yes vs. no)	0.418	0.357	0.030	4.309
Relapsed disease (yes vs. no)	0.999	0.000	0.000	–
Bone marrow blasts (≥5% vs. <5%)	0.999	–	0.000	–
Extramedullary disease (yes vs. no)	0.711	0.625	0.052	7.530
CNSL (yes vs. no)	0.920	0.880	0.072	10.753
BCR/ABL1 (positive vs. negative)	0.920	0.880	0.072	10.753
SH2B3 mutation (positive vs. negative)	0.321	0.283	0.023	3.425
PAX5 mutation (positive vs. negative)	0.236	0.220	0.018	2.688
WBC ( ≥ 30 × 10^9^/L vs. <30 × 10^9^/L)	0.998	0.000	0.000	–
Lymphodepletion regimens (Flu + Cy vs. non-Flu + Cy)	0.920	0.880	0.072	10.753
CAR-T cells (anti-CD19 + CD22 vs. anti-CD19)	0.277	0.250	0.021	3.041
Multivariate logistic regression				
No independent factor	–	–	–	–

Factors affecting CR were determined by univariate and multivariate logistic regression analysis with Forward Stepwise (Conditional) method. *P* value <0.05 was considered significant.

*CR* complete remission, *CNSL* central nervous system leukemia, *WBC* white blood cell, *OR* odds ratio, *CI* confidence interval, *Flu* fludarabine, *Cy* cyclophosphamide, *CAR-T* chimeric antigen receptor T cells.

### Survival profiles by CAR-T therapy

**Fig. 2:**
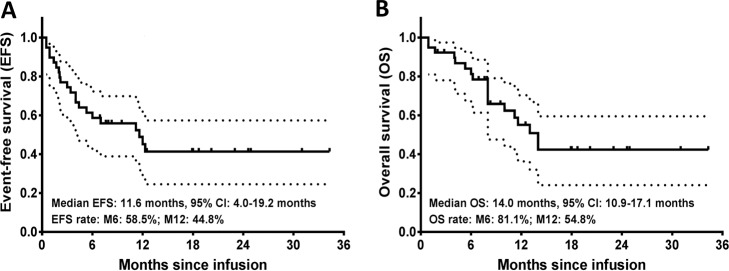
Survival profiles by CAR-T therapy in R/R B-ALL patients. The median EFS was 11.6 months, and the possibility of EFS was 58.5% at M6 and 44.8% at M12 (**a**). The median OS was 14.0 months, and the possibility of OS was 81.1% at M6 and 54.8 at M12 (**b**). Kaplan–Meier curve was used to demonstrate survival profiles. EFS, event-free survival; OS, overall survival; M6, 6 months; M12, 12 months.

The median EFS was 11.6 months (95% confidence interval (CI): 4.0–19.2 months) with 6-month EFS being 58.5% and 1-year EFS being 44.8% (Fig. 2a). Additionally, the median OS was 14.0 months (95% CI: 10.9–17.1 months) with 6-month OS being 81.1% and 1-year OS being 54.8% (Fig. 2b).

### Factors affecting EFS by CAR-T therapy

**Fig. 3:**
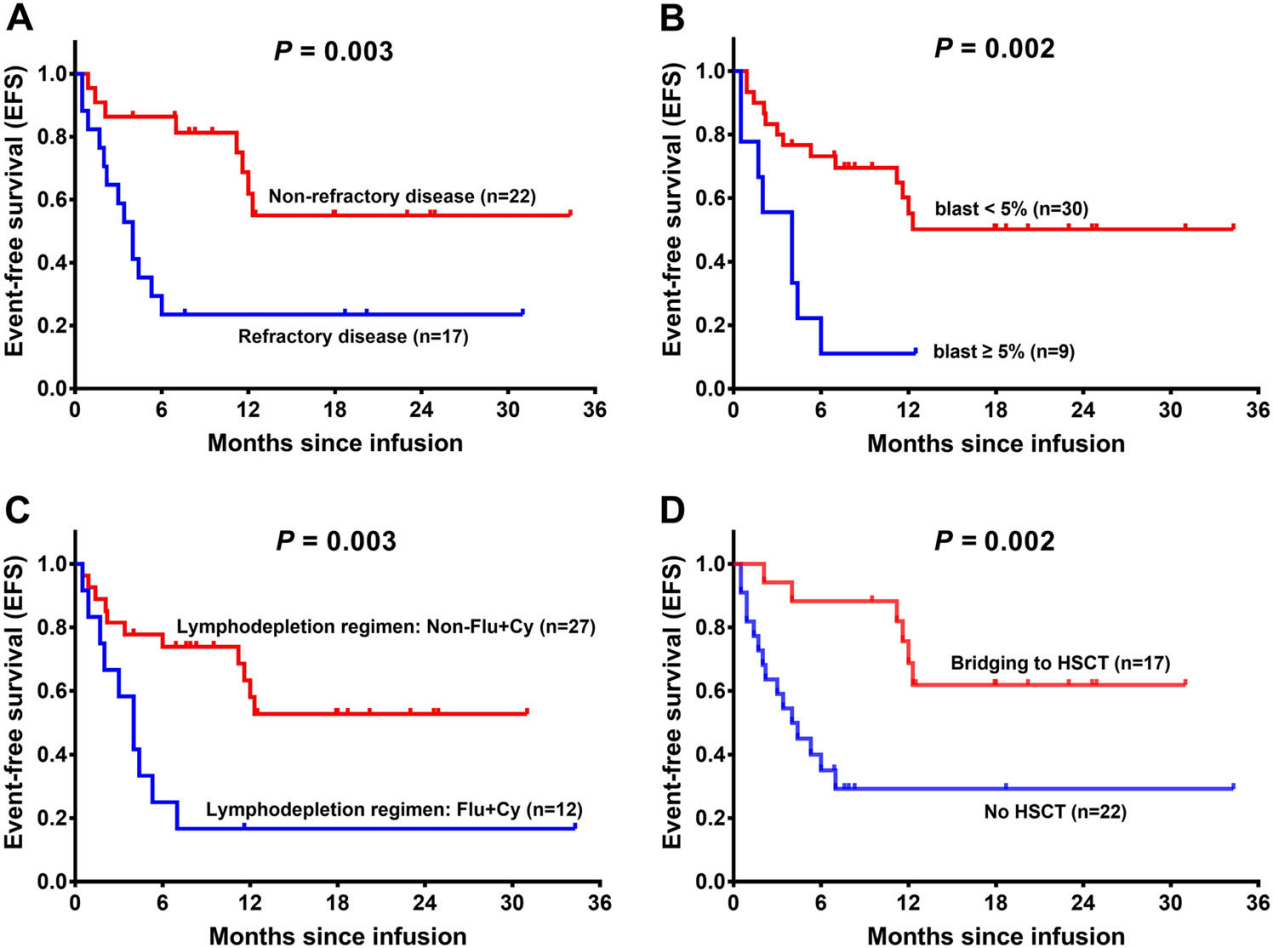
Subgroup analysis of EFS by CAR-T therapy in R/R B-ALL patients (statistically significant). Better EFS was observed in patients with non-refractory disease compared with patients with refractory disease (**a**), in patients with blast <5% compared with patients with blast ≥5% (**b**), in patients with other lymphodepletion regimens compared with patients with Flu + Cy regimen (**c**), as well as in patients bridged to HSCT compared with patients with CAR-T therapy only (**d**). Kaplan–Meier curve was used to demonstrate survival profiles, and the log-rank test was used to determine the difference between paired subgroups. *P* < 0.05 was considered significant. EFS, event-free survival; Flu + Cy, cyclophosphamide plus fludarabine; HSCT, hematopoietic stem-cell transplantation; CAR-T, chimeric antigen-specific T cell.

Patients were divided into subgroups depending on their baseline characteristics and treatment regimens, and subgroup analysis disclosed that EFS was reduced in patients with refractory disease (*P* = 0.003) (Fig. 3a), elevated bone marrow blast (*P* = 0.002) (Fig. 3b), Flu + Cy lymphodepletion regimen (*P* = 0.003) (Fig. 3c), and no HSCT (*P* = 0.002) (Fig. 3d). No correlation of other characteristics with EFS was observed (all *P* > 0.05) (Supplementary Fig. 1). Univariate Cox’s regression model illustrated that refractory disease (yes vs. no) (hazard ratio (HR) = 3.528, *P* = 0.006), bone marrow blasts (≥5 vs. <5%) (HR = 3.768, *P* = 0.004), lymphodepletion regimens (Flu + Cy vs. non-Flu + Cy) (HR = 3.492, *P* = 0.006) predicted shorter EFS, while bridging to HSCT (yes vs. no) (HR = 0.229, *P* = 0.004) was associated with longer EFS (Table 4). Multivariate Cox’s regression analysis further exhibited that bone marrow blasts (≥5 vs. <5%) (HR = 3.259, *P* = 0.013) was an independent risk factor for worse EFS, whereas bridging to HSCT (yes vs. no) (HR = 0.247, *P* = 0.008) independently predicted better EFS in R/R B-ALL patients underwent CAR-T therapy.

**Table 4:** Factors affecting EFS by Cox’s regression analysis.

Items	Cox’s regression model			
	*P* value	HR	95% CI	
			Lower	Higher
Univariate Cox’s regression				
Age (≥18 vs. <18 years)	0.624	0.789	0.305	2.039
Gender (male vs. female)	0.876	0.934	0.393	2.218
Number of previous chemotherapies (≥4 vs. <4)	0.513	1.340	0.558	3.218
Refractory disease (yes vs. no)	0.006	3.528	1.438	8.659
Relapsed disease (yes vs. no)	0.150	0.399	0.114	1.393
Bone marrow blasts (≥5% vs. <5%)	0.004	3.768	1.521	9.334
Extramedullary disease (yes vs. no)	0.570	0.780	0.331	1.840
CNSL (yes vs. no)	0.104	0.401	0.133	1.207
BCR/ABL1 (positive vs. negative)	0.108	0.431	0.154	1.203
SH2B3 mutation (positive vs. negative)	0.604	0.786	0.317	1.952
PAX5 mutation (positive vs. negative)	0.319	0.600	0.220	1.640
WBC (≥30 × 10^9^/L vs. <30 × 10^9^/L)	0.455	1.390	0.585	3.304
Lymphodepletion regimens (Flu + Cy vs. non-Flu + Cy)	0.006	3.492	1.437	8.484
CAR-T cells (anti-CD19 + CD22 vs. anti-CD19)	0.118	2.142	0.823	5.574
CR with MRD negative (yes vs. no)	0.094	0.456	0.182	1.144
Bridging to HSCT (yes vs. no)	0.004	0.229	0.084	0.623
Multivariate Cox’s regression				
Bone marrow blasts (≥5% vs. <5%)	0.013	3.259	1.283	8.282
Bridging to HSCT (yes vs. no)	0.008	0.247	0.088	0.695

Factors affecting EFS were determined by univariate and multivariate Cox’s proportional hazards regression model with Forward Stepwise (Conditional) method. *P* value <0.05 was considered significant.

*EFS* event-free survival, *CR* complete remission, *MRD* minimal residual disease, *CNSL* central nervous system leukemia, *WBC* white blood cell, *Flu* fludarabine, *Cy* cyclophosphamide, *CAR-T* chimeric antigen receptor T cells, *HSCT* hematopoietic stem-cell transplantation, *HR* hazard ratio, *CI* confidence interval.

### Factors affecting OS by CAR-T therapy

**Fig. 4:**
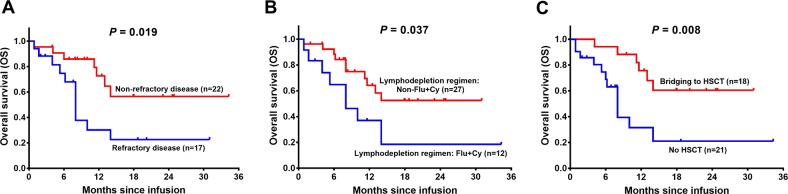
Subgroup analysis of OS by CAR-T therapy in R/R B-ALL patients (statistically significant). OS was longer in patients with non-refractory disease compared with patients with refractory disease (**a**), patients with other lymphodepletion regimens compared with patients with Flu + Cy regimen (**b**) and in patients with bridging to HSCT compared with patients with CAR-T therapy only (**c**). Kaplan–Meier curve was used to demonstrate survival profiles, and the log-rank test was used to determine the difference between paired subgroups. *P* < 0.05 was considered significant. OS, overall survival; Flu + Cy, cyclophosphamide plus fludarabine; HSCT, hematopoietic stem-cell transplantation; CAR-T, chimeric antigen-specific T cell.

As for subgroup analysis of OS, patients with refractory disease (*P* = 0.019) (Fig. 4a), Flu + Cy lymphodepletion regimen (*P* = 0.037) (Fig. 4b), and no HSCT (*P* = 0.008) (Fig. 4c) were with worse OS, while other characteristics was not correlated with OS (all *P* > 0.05) (Supplementary Fig. 2). Univariate Cox’s regression analysis displayed that refractory disease (yes vs. no) (HR = 2.914, *P* = 0.028) and lymphodepletion regimens (Flu + Cy vs. non-Flu + Cy) (HR = 2.574, *P* = 0.049) were associated with poor OS, while bridging to HSCT (yes vs. no) (HR = 0.279, *P* = 0.014) predicted longer OS (Table 5). Further multivariate Cox’s regression disclosed that bridging to HSCT (yes vs. no) (HR = 0.279, *P* = 0.014) was an independent protective factor for better OS in R/R B-ALL patients who underwent CAR-T therapy.

**Table 5:** Factors affecting OS by Cox’s regression analysis.

Items	Cox’s regression model			
	*P* value	HR	95% CI	
			Lower	Higher
Univariate Cox’s regression				
Age (≥18 vs. <18 years)	1.000	1.000	0.374	2.673
Gender (male vs. female)	0.850	0.914	0.359	2.324
Number of previous chemotherapies (≥4 vs. <4)	0.996	0.997	0.393	2.531
Refractory disease (yes vs. no)	0.028	2.914	1.122	7.571
Relapsed disease (yes vs. no)	0.199	0.365	0.079	1.697
Bone marrow blasts (≥5% vs. <5%)	0.065	2.557	0.945	6.921
Extramedullary disease (yes vs. no)	0.529	0.743	0.295	1.874
CNSL (yes vs. no)	0.084	0.333	0.096	1.158
BCR/ABL1 (positive vs. negative)	0.249	0.539	0.188	1.541
SH2B3 mutation (positive vs. negative)	0.198	0.480	0.157	1.468
PAX5 mutation (positive vs. negative)	0.167	0.417	0.120	1.444
WBC (≥30 × 10^9^/L vs. <30 × 10^9^/L)	0.695	1.205	0.474	3.066
Lymphodepletion regimens (Flu + Cy vs. non-Flu + Cy)	0.049	2.574	1.005	6.598
CAR-T cells (anti-CD19 + CD22 vs. anti-CD19)	0.263	1.925	0.611	6.059
CR with MRD negative (yes vs. no)	0.080	0.411	0.152	1.111
Bridging to HSCT (yes vs. no)	0.014	0.279	0.101	0.769
Multivariate Cox’s regression				
Bridging to HSCT (yes vs. no)	0.014	0.279	0.101	0.769

Factors affecting OS were determined by univariate and multivariate Cox’s proportional hazards regression model with Forward Stepwise (Conditional) method. *P* value <0.05 was considered significant.

*OS* overall survival, *CR* complete remission, *MRD* minimal residual disease, *CNSL* central nervous system leukemia, *WBC* white blood cell, *Flu* fludarabine, *Cy* cyclophosphamide, *CAR-T* chimeric antigen receptor T cells, *HSCT* hematopoietic stem-cell transplantation, *HR* hazard ratio, *CI* confidence interval.

### Adverse events by CAR-T therapy and management for CRS

After treatment with CAR-T, the incidences of adverse events were recorded, and we found that 38 (97.4%) patients developed CRS and the rates of grade I, grade II, grade III, grade IV, and grade V (death) CRS were 51.3%, 7.7%, 15.4%, 20.5%, and 2.6% respectively (Table 6). The common symptoms (incidences) of CRS included fever (71.8%), hypotension (51.3%), hypoxemia (28.2%), capillary leak syndrome (30.8%), hepatic injury (53.8%), and heart injury (12.8%). Other adverse events included hematologic toxicities (grade I: 5.1%, grade II: 7.7%, grade III: 17.9%, grade IV: 69.2%), neurotoxicity (28.2%), infection (38.5%), and admission for ICU (10.3%). As for CRS management, 12 (30.8%) patients received glucocorticoids and 15 (38.5%) patients were treated with tocilizumab injection.

**Table 6:** Adverse events to CAR-T and treatments for CRS.

Items	Patients (*n* (%))
CRS	38 (97.4)
Grade I	20 (51.3)
Grade II	3 (7.7)
Grade III	6 (15.4)
Grade IV	8 (20.5)
Grade V	1 (2.6)
Symptoms of CRS	
Fever	28 (71.8)
Hypotension	20 (51.3)
Hypoxemia	11 (28.2)
Capillary leak syndrome	12 (30.8)
Hepatic injury	21 (53.8)
A	12 (30.8)
B	5 (12.8)
C	4 (10.3)
Heart injury	5 (12.8)
I	4 (10.3)
IV	1 (2.6)
Hematologic toxicities grade	
I	2 (5.1)
II	3 (7.7)
III	7 (17.9)
IV	27 (69.2)
Neurotoxicity	11 (28.2)
Infection	15 (38.5)
Admission for ICU	4 (10.3)
Treatments for CRS	
Glucocorticoids	12 (30.8)
Tocilizumab injection	15 (38.5)

Data were presented as count (percentage).

*CAR-T* chimeric antigen receptor T cells, *CRS* cytokine release syndrome.

### Factors affecting severe CRS by CAR-T therapy

The incidence of severe CRS was 38.5%, and univariate logistic regression analysis exhibited that refractory disease (yes vs no) (odds ratio (OR) = 8.250, *P* = 0.005) was correlated with higher risk of severe CRS (Table 7). In addition, multivariate logistic regression analysis showed that refractory disease (yes vs. no) (OR = 12.599, *P* = 0.005) independently predicted high occurrence of severe CRS, while WBC (≥30 × 10^9^/L vs. <30 × 10^9^/L) (OR = 0.169, *P* = 0.047) independently predicted decreased occurrence of severe CRS in R/R B-ALL patients who underwent CAR-T therapy.

**Table 7:** Factors affecting occurrence of severe CRS by logistic regression analysis.

Items	Logistic regression model			
	*P* value	OR	95% CI	
			Lower	Higher
Univariate logistic regression				
Age (≥18 vs. <18 years)	0.096	3.325	0.809	13.674
Gender (male vs. female)	0.721	1.269	0.343	4.696
Number of previous chemotherapies (≥4 vs. <4)	0.759	0.816	0.223	2.992
Refractory disease (yes vs. no)	0.005	8.250	1.895	35.910
Relapsed disease (yes vs. no)	0.148	0.174	0.016	1.857
Bone marrow blasts (≥5% vs. <5%)	0.675	1.382	0.305	6.255
Extramedullary disease (yes vs. no)	0.334	0.525	0.142	1.943
CNSL (yes vs. no)	0.334	0.525	0.142	1.943
BCR/ABL1 (positive vs. negative)	0.256	0.417	0.092	1.888
SH2B3 mutation (positive vs. negative)	0.171	0.350	0.078	1.573
PAX5 mutation (positive vs. negative)	0.236	0.430	0.106	1.739
WBC (≥30 × 10^9^/L vs. <30 × 10^9^/L)	0.082	0.300	0.077	1.163
Lymphodepletion regimens (Flu + Cy vs. non-Flu + Cy)	0.784	1.214	0.303	4.867
CAR-T cells (anti-CD19 + CD22 vs. anti-CD19)	0.792	0.833	0.215	3.230
Multivariate logistic regression				
Refractory disease (yes vs. no)	0.005	12.599	2.187	72.587
WBC (≥30 × 10^9^/L vs. <30 × 10^9^/L)	0.047	0.169	0.029	0.978

Factors affecting occurrence of severe CRS were determined by univariate and multivariate Logistic regression analysis with Forward Stepwise (Conditional) method. *P* value <0.05 was considered significant.

*CRS* cytokine release syndrome, *CR* complete remission, *MRD* minimal residual disease, *CNSL* central nervous system leukemia, *WBC* white blood cell, *Flu* fludarabine, *Cy* cyclophosphamide, *CAR-T* chimeric antigen receptor T cells, *OR* odds ratio, *CI* confidence interval.

### Adverse events by HSCT

There were 17 patients bridged to HSCT after CAR-T therapy and the occurrences of adverse events by HSCT were recorded (Table 8). The incidences of GVHD, erythra, gastrointestinal reactions, hepatic injury, hemorrhage cystitis, hepatic veno-occlusive disease, and infection were 64.7%, 35.3%, 23.7%, 23.7%, 5.9%, 5.9%, and 88.2%, respectively, in R/R B-ALL patients underwent CAR-T and bridged to HSCT.

**Table 8:** Adverse events after HSCT.

Adverse events	Patients (*n*/%)
GVHD	11/17 (64.7)
I	2/17 (11.8)
II	5/17 (29.4)
IV	4/17 (23.5)
Erythra	6/17 (35.3)
Gastrointestinal reactions	4/17 (23.7)
Hepatic injury	4/17 (23.7)
Hemorrhagic cystitis	1/17 (5.9)
Hepatic veno-occlusive disease	1/17 (5.9)
Infection	15/17 (88.2)

Data were presented as count (percentage).

*CAR-T* chimeric antigen receptor T cells, *GVHD* graft vs. host disease, *HSCT* hematopoietic stem-cell transplantation.

## Discussion

In Chinese R/R B-ALL patients who underwent CAR-T therapy: (1) 92.3% cases achieved CR and 76.9% cases achieved CR with MRD negative to CAR-T therapy; (2) the median EFS and OS were 11.6 and 14.0 months, respectively; (3) refractory disease and high bone marrow blast level were independent risk factors for both EFS and OS; (4) the incidence of CRS was 97.4%, and other common adverse events included hematologic toxicities, neurotoxicity, infection, and admission for ICU.

The normal adaptive immune system is capable of recognizing and destructing cancerous cells by the following approaches. Tumor-associated antigen is presented by antigen-presenting cells (APCs) that are matured from dendric cells, and IL-12 is released to activate type I CD4^+^ T helper cells. The CD4^+^ T cells are then stimulated and recognize antigen-presenting MHC on APC along with co-stimulatory proteins, including CD28 and 4-1BB. Subsequently, cytotoxic T cells are activated and release perforin as well as granzyme B that induce apoptosis of malignant cells^11,22^. However in B-ALL, which is characterized by cancerous progression that takes place in B cell lineage of immature lymphoid cells, leukemic B cells reduce the expression of MHC and upregulate immunosuppression cells to escape from the adaptive immune system^3^. The purpose of CAR-T in B-ALL is to enable tumor-associated antigen recognition of T cells regardless of MHC, and the mechanism is to develop a CAR construct, which contains scFv extracellularly (for antigen binding), transmembrane domain (for receptor stabilization), and CD3ζ signaling domain intracellularly (for T cell stimulation)^23^. On the basis of the first-generation CAR (only scFv and CD3ζ), the second-generation CAR adds co-stimulatory signaling domain such as CD28 and 4-1BB, which improves proliferation and persistence of T cells. The third-generation CAR adds two or more co-stimulatory signaling domains intracellularly^21^.

In clinical trials, the second-generation CAR constructs are most commonly used for CAR-T therapy. The processes of CAR-T therapy can be simplified as: obtaining autologous T cells by leukapheresis; selection of cytotoxic T cells; transduction using viral vector; replication and extension of CAR-T cells; conditioning chemotherapy; CAR-T cell infusion^24^. In this current study, lentiviral vector instead of retrovirus was used for transduction due to its stability, wide spectrum of infection, and less carcinogenicity. In addition, lymphodepletion regimens were given to the patients prior to CAR-T infusion in order to reduce the homeostatic regulation upon T cells, and the lymphopenic environment has been reported to favor adoptive T cell transfer and improve the effectiveness of CAR-T therapy^25,26^.

CAR-T therapy has been illustrated to present satisfying treatment response in R/R B-ALL patients by some clinical trials with limited samples. Park et al.^9^ harness lentivirally transduced CD19-CAR-T cells with CD3ζ and CD28 to treat R/R B-ALL, and report that 83% of patients achieve CR and 67% of patients achieve CR and remain MRD negative. A phase I/IIA trial by Maude et al.^27^ exhibits that in R/R B-ALL patients treated with lentivirally transduced CD19-CAR-T cells with CD3ζ and 4-1BB, the CR rate is 90% and the rate of MRD negativity is 73%. Also, another phase II single cohort and multicenter clinical trial by Laetsch and colleagues^28^ using lentivirally transduced CD19-CAR-T cells with CD3ζ and 4-1BB discloses that the CR rate is 60% and the rate of CR with incomplete hematologic recovery is 21%, summing up to an overall remission rate of 81% in patients with R/R B-ALL^28^. These previous clinical trials, based on the United States and Europe, show satisfying remission rates in response to lentivirally transduced CD19-CAR-T cells in R/R B-ALL patients, while these trials are based on white population in the United States and Europe, and their sample sizes are relatively small. As for studies in China, there is only one published study, which recruits 30R/R B-ALL patients for CAR-T therapy and discloses that the CR is 86.67% and CR with MRD negative is 83.33%; however, the sample size is still relatively small, and the survival information by CAR-T therapy is lacking^29^. In this present study, we observed that the CR rate to CAR-T therapy was 92.3% and CR with MRD-negative rate was 76.9% in 39R/R B-ALL patients, whose values were slightly higher compared to the previous clinical trials. This could be attributed to that the proportion of younger patients in this study might be larger than the other two studies (69% of R/R B-ALL patients were juveniles in our study). Due to the difference in study design, patients’ characteristics, treatment regimens, and so on, the horizontal comparison of CR rates among different studies is of limited meaning, while our data could still imply that CAR-T therapy achieved promising treatment response in Chinese R/R B-ALL patients.

There are several factors that predict poor treatment response to conventional chemotherapies in ALL patients, such as prior chemotherapy regimens and transplantation history^12^. However, as for factors influencing treatment response to CAR-T therapy in R/R B-ALL patients, only a few small-scale studies are reported, which illuminate no association between conventional risk factors and CR to CAR-T therapy in R/R B-ALL patients^27,30^. Considering that there are no study investigating risk factors for treatment response in Chinese R/R B-ALL patients, we reviewed the medical records of R/R B-ALL patients who underwent CAR-T therapy and evaluated the correlation of patients’ characteristics and treatment regimens with CR. In accordance with the previous clinical trials, we discovered that none of the characteristics selected in our study was associated with CR to CAR-T therapy in Chinese R/R B-ALL patients. This might be due to that the number of patients included in this study was not enough and over 90% of patients achieved CR, which led to the limited event of non-CR. Due to the absence of effective event, no statistically significant predictive factor for CR was observed in our analysis. Therefore, it is necessary to evaluate the predictive factors for treatment response to CAR-T therapy in R/R B-ALL patients with larger sample size.

The survival benefit is one of the indispensable assessments for treatment efficacy in hematological malignancies; however, only a few studies have conducted the evaluation of survival profiles for CAR-T therapy in R/R B-ALL patients, which is as a result of the application of CAR-T in R/R B-ALL that has just started. In the study of Park et al.^9^, the median EFS is 6.1 months and the median OS is 12.9 months in R/R B-ALL patients. A study by Maude et al.^27^ discloses that the 6-month EFS rate is 67% and the 6-month OS rate is 78% in R/R B-ALL patients treated with CD19-CAR-T cells with CD3ζ and 4-1BB. Moreover, a median EFS of 7.6 months and a median OS of 20.0 months have been reported by Hay et al.^31^ in adult R/R B-ALL patients achieving MRD-negative CR after CD19-CAR-T cell therapy. While in China no study investigating the survival profiles by CAR-T has been reported, it is very critical to explore the effect of CAR-T therapy on survival in order to improve the overall prognosis of R/R B-ALL patients. Therefore, this present study investigated the survival profiles by CAR-T therapy in Chinese R/R B-ALL patients. The median EFS was 11.6 months with the 6-month EFS being 58.5% and the 12-month EFS being 44.8%; the median OS was 14.0 months with the 6-month OS being 81.1% and the 12-month OS being 54.8%. In this present study, the values of EFS and OS by CAR-T therapy in R/R B-ALL patients were similar or slightly higher than that in previous studies. This might be attributed to that: (1) Our study included a larger proportion of juvenile R/R B-ALL patients (up to 69% patients were under 18 years) than that in previous studies, who might respond more positively to the treatment and present longer survival compared with the elder patients. (2) The follow-up duration was relatively longer in our study (median: 9.5 months; range: 0–34.3 months) compared to most previous studies; thus, the EFS and OS was numerically longer. Our study was the first to reveal the survival profiles of CAR-T therapy in Chinese R/R B-ALL patients, which provided valuable reference for wide application of CAR-T therapy in China.

The individual treatment is critical for R/R B-ALL therapy, and for CAR-T therapy, it is also essential to explore predictive factors for survival profiles in R/R B-ALL patients to improve patients’ prognosis. Park et al.^9^ illustrate that patients with lower disease burden have longer EFS and OS than those with heavy disease burden; in addition, the ratio of CAR-T cells to disease burden rather than CAR-T cell dose or peak CAR-T cell expansion is correlated with better EFS and OS. Besides, Turtle et al.^32^ report that the addition of Flu to cyclophosphamide as lymphodepletion regimen prior to CAR-T infusion improves disease-free survival in patients with R/R B-ALL. In addition, bridging to HSCT is also a strong contributor to better EFS as reported by Hay et al.^31^. These studies suggest that the consolidation strategies, such as HSCT, might benefit with reducing relapse of R/R B-ALL. However, the aforementioned studies are mainly carried out in the United States and Europe, which are based on the white population. Furthermore, there is still no study in China that reports the predictive factors for survival profiles in R/R B-ALL patients. Thus, in the current study, we analyzed factors before and during CAR-T treatment that might influence the survival profiles by CAR-T therapy in Chinese R/R B-ALL patients and discovered that reduced bone marrow blast and bridging to HSCT independently predicted longer EFS, and bridging to HSCT was an independent protective factor for OS. The possible explanations were: (1) No refractory disease and reduced bone marrow blast meant less disease severity, which might lead to reduced ALL-related symptoms and contribute to better survival. (2) Patients who received HSCT have restored or partially restored hematopoietic system and immune system, which could favor the prognosis, especially the long-term survival.

As a cellular therapy for B-ALL, CAR-T is highly effective, while it also inevitably results in toxicities, including CRS (a systemic cytokine process by proliferation and activation of T cells) and neurotoxicity^17^. CRS is classified into five stages (the fifth stage being death) according to severity and management requirements, and CRS above (including) stage 3 was considered severe and life-threatening^21^. According to previous studies, most of the R/R B-ALL patients develop CRS in response to CAR-T therapy and the incidence of severe CRS is around 30%^28,30^, but a smaller incidence is reported in younger patients^33^. In our study, 97.4% of patients developed CRS and the rate of severe CRS was 38.5%, which was in accordance with the previous researches, which supported the safe use of CAR-T therapy in Chinese R/R B-ALL patients. However, numerically, the incidence of severe CRS was higher in this study compared with that of pediatric/young adults^33^, which might be due to the inclusion of elder patients or the insufficient samples. Furthermore, predictive markers for CRS have been increasingly described by clinical studies in requirement of toxicity management for CAR-T therapy^34–36^. It is revealed that the degrees of CAR-T cell expansion and disease burden are positively correlated with severity of CRS, and high levels of several laboratory markers, including C-reactive protein, ferritin, and membrane cofactor protein (MCP), have been discovered to indicate increased CRS risk^34,36^. Additionally, bulk CD8^+^ cell selection and lymphodepletion/CAR-T dose interaction (which means that the lymphodepletion regimen more greatly associates with CRS as CAR-T dose increases) are correlated with elevated risk of CRS^34^. However, patient-specific characteristics, including age, previous therapy, genetic risk profile, and CAR-T cell dose, present no association with CRS occurrence^35^. Each of the previous studies reveal several risk factors for CRS, whereas studies that systematically evaluate factors influencing severe CRS are still rare in China. In this study, we used logistic regression analysis to determine factors affecting severe CRS, and observed that refractory disease independently predicted increased incidence of severe CRS and greater WBC (≥30 × 10^9^/L vs. <30 × 10^9^/L) independently predicted reduced risk of severe CRS, whereas other patient’s characteristics had no association with severe CRS occurrence, which to some extent consisted with the previous evidences. The possible reasons were that: (1) the activation of CAR-T cells was driven by recognition and binding to CD19 on malignant B cells; therefore, heavier disease burden might lead to higher level of CD19-expressing B cells, which possibly promoted activation of CAR-T cells as well as increased secretion of cytokines, thereby raising the risk of severe CRS in R/R B-ALL patients. (2) Depletion of immune cells was a common complication of CAR-T therapy, and higher degree of immune depletion (B cell aplasia), on the other hand, indicated the better effect of CAR-T cells on killing B cells^37^. Therefore, greater WBC suggested less depletion of immune cells, which might implicate that CAR-T cell activation and the subsequent release of cytokines were decreased, and thereby the incidence of severe CRS was lower. Except for CRS, existing evidences also exhibit other common adverse events by CAR-T therapy, including fever, neurologic side effects, and infection due to B cell aplasia, and so on^12,38^. Our study observed that the common adverse events, except CRS induced by CAR-T therapy in Chinese R/R B-ALL patients, included hematologic toxicities, neurotoxicity, infection, and admission for ICU, which were in line with the anticipation of previous evidences. The safety profiles by CAR-T in our study implied that CAR-T was relatively well tolerated in Chinese R/R B-ALL patients with expectable and manageable adverse effect.

In this study, there still remained several limitations. Firstly, the scale of this study was still small due to limited cases and single hospital base, which was not powered for moderate outcomes. However, the sample size in this present study was relatively greater than that in most clinical trials previously reported, and more patients were to be included in the future. Secondly, the patient composition in our study was juveniles (69%) plus adults (31%), while elder patients (≥65 years) were not included. Thus, the efficacy and safety of CAR-T therapy in elder patients needed to be further investigated. Additionally, clinical challenges for CAR-T therapy still exist in reducing major complications, limited or over-activated proliferation of adoptive T cells after infusion, coping with CD19-negative relapse, high expenses, and so on.

In conclusion, CAR-T therapy presents a promising treatment efficacy by inducing high CR (92.3%) and CR with MRD-negative (76.9%) rates as well as achieving median EFS and OS of 11.6 and 14.0 months, respectively, and its safety profiles is also tolerable in Chinese R/R B-ALL patients. Besides, we found that refractory disease and high bone marrow blast level independently predicted worse survival, and refractory disease as well as low WBC was independent risk factors for severe CRS, which provides more evidence for future individual treatment of R/R B-ALL involving CAR-T therapy.

## Supplementary information

Supplementary Figure Legends

Supplementary Figure 1

Supplementary Figure 2

Detailed Attribution of Authorship

Reproducibility Checklist
