# Supplementary material for: Treatment response, survival, safety, and predictive factors to chimeric antigen receptor T cell therapy in Chinese relapsed or refractory B cell acute lymphoblast leukemia patients
Source: Cell Death Dis. 2020 Mar 30;11(3):207. doi: 10.1038/s41419-020-2388-1 (PMC7105502; doi:10.1038/s41419-020-2388-1)
Supplement: Supplementary file 1 — Supplementary Figure Legends [file 41419_2020_2388_MOESM1_ESM.docx]

**Supplementary Figure 1.** Subgroup analysis of EFS by CAR-T therapy in R/R B-ALL patients (statistically non-significant). No difference in EFS was observed in subgroups including: age≥18 years vs age <18 years (**A**); male vs female (**B**); number of previous chemotherapies ≥4 vs number of previous chemotherapies <4 (**C**); relapse disease vs non-relapse disease (**D**); extramedullary disease vs non-extramedullary disease (**E**); CNSL vs non-CNSL (**F**); BCR-ABL-positive vs BCR-ABL-negative (**G**); SH2B3-positive vs SH2B3-negative (**H**); PAX5-positive vs PAX5-negative (**I**); WBC ≥30 x10^9^/L vs WBC <30 x10^9^/L (**J**); Anti-CD19+CD22 vs Anti-CD19 (**K**); CR with MRD positive vs CR with MRD negative (**L**). Kaplan–Meier curve was used to demonstrate survival profiles, and the log-rank test was used to determine the difference between paired subgroups. *P* <0.05 was considered significant. EFS, event free survival; CNSL, central nervous system leukemia; WBC, white blood cell; CR, complete remission; MRD, minimal residual disease.

**Supplementary Figure 2.** Subgroup analysis of OS by CAR-T therapy in R/R B-ALL patients (statistically non-significant). No difference in OS was observed in subgroups including: age≥18 years vs age <18 years (**A**); male vs female (**B**); number of previous chemotherapies ≥4 vs number of previous chemotherapies <4 (**C**); relapse disease vs non-relapse disease (**D**); bone marrow blast ≥5% vs bone marrow blast <5% (**E**); extramedullary disease vs non-extramedullary disease (**F**); CNSL vs non-CNSL (**G**); BCR-ABL-positive vs BCR-ABL-negative (**H**); SH2B3-positive vs SH2B3-negative (**I**); PAX5-positive vs PAX5-negative (**J**); WBC ≥30 x10^9^/L vs WBC <30 x10^9^/L (**K**); Anti-CD19+CD22 vs Anti-CD19 (**L**); CR with MRD positive vs CR with MRD negative (**M**). Kaplan–Meier curve was used to demonstrate survival profiles, and the log-rank test was used to determine the difference between paired subgroups. *P* <0.05 was considered significant. OS, overall survival; CNSL, central nervous system leukemia; WBC, white blood cell; CR, complete remission; MRD, minimal residual disease.
