# Supplementary figures and images for: Treatment response, survival, safety, and predictive factors to chimeric antigen receptor T cell therapy in Chinese relapsed or refractory B cell acute lymphoblast leukemia patients
Source: Cell Death Dis. 2020 Mar 30;11(3):207. doi: 10.1038/s41419-020-2388-1 (PMC7105502; doi:10.1038/s41419-020-2388-1)

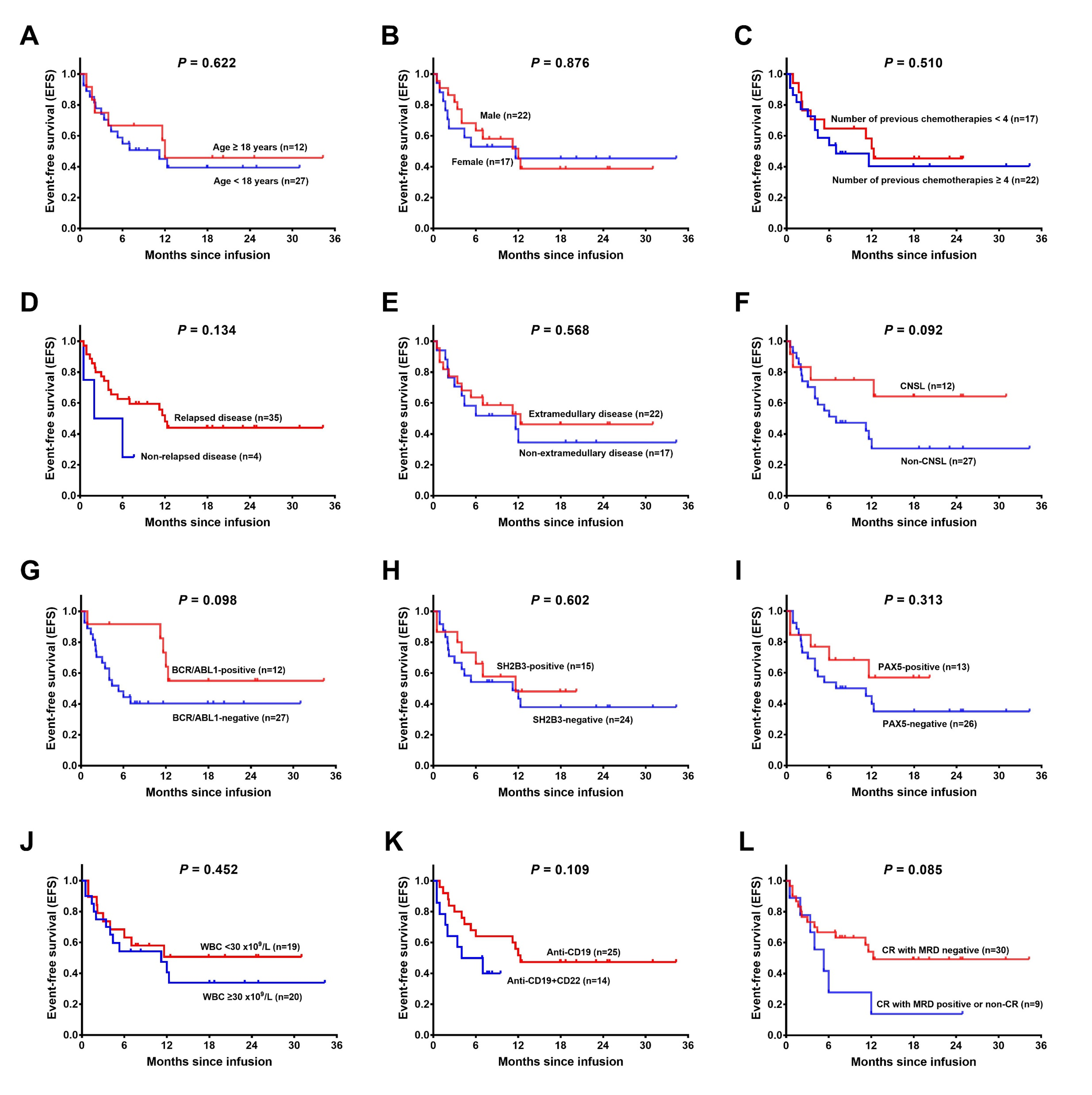

Supplement: Supplementary file 2 — Supplementary Figure 1 [file 41419_2020_2388_MOESM2_ESM.tif]

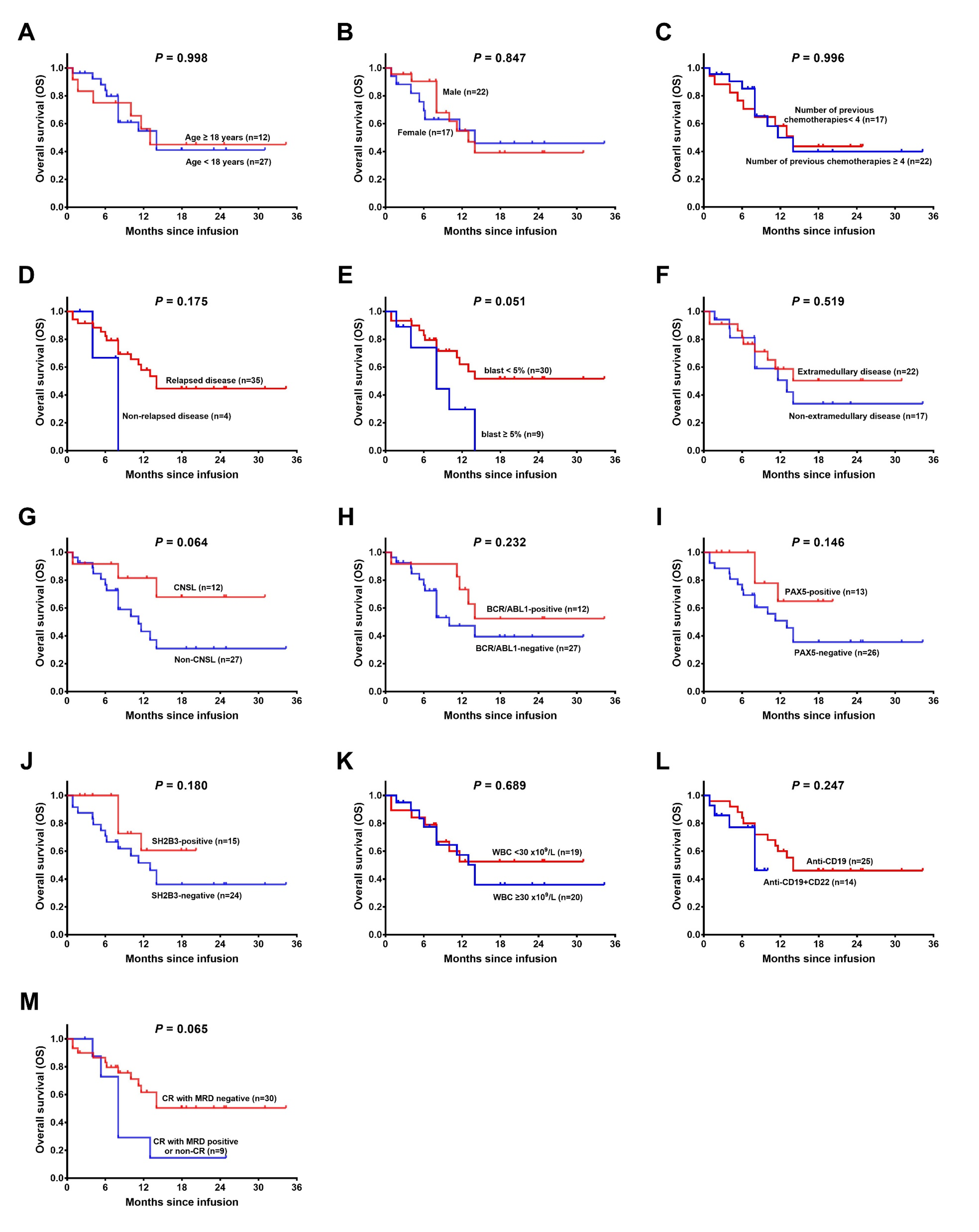

Supplement: Supplementary file 3 — Supplementary Figure 2 [file 41419_2020_2388_MOESM3_ESM.tif]
