# Supplementary material for: Treatment response, survival, safety, and predictive factors to chimeric antigen receptor T cell therapy in Chinese relapsed or refractory B cell acute lymphoblast leukemia patients
Source: Cell Death Dis. 2020 Mar 30;11(3):207. doi: 10.1038/s41419-020-2388-1 (PMC7105502; doi:10.1038/s41419-020-2388-1)
Supplement: Supplementary file 4 — Detailed Attribution of Authorship [file 41419_2020_2388_MOESM4_ESM.pdf]

DECLARATION OF CONTRIBUTIONS TO ARTICLE

ADMC

Manuscript Number: CDDIS-19-4215R

Journal Name: *Cell Death & Disease* (the 'Journal')

Proposed Title of the Contribution:

Circ_0001955 facilitates hepatocellular carcinoma (HCC) tumorigenesis by (the 'Contribution)

Author(s):

Limin Li, Jie Liu, Mengyuan Xu, Hongjuan Yu, Chengfang Lv, Fenglin Cao, Zhenkun Wang, Yueyue Fu, Mingwen Zhang, Hongbin Meng, Xiaoqian Zhang, Liqing Kang, Zhuo Zhang, Jinmei Li, Jiawei Feng, Xin Lian, Lei Yu, Jin Zhou (the 'Authors')

For all *CDDis* articles, each person named as an author in the published version must be able to show he or she has contributed substantially to the article.

Authorship credit should be based on 1) substantial contributions to conception and design, acquisition of data, or analysis and interpretation of data; 2) drafting the article or revising it critically for important intellectual content; and 3) final approval of the version to be published. Authors should meet conditions 1, 2 and 3.

Any person who cannot be shown to have made a substantial contribution to the article cannot be listed as an author in the final version. The name of any person who is deemed to have made a minor contribution can, however, appear in the Acknowledgments section of the article.

Please complete the table below to indicate the contributions of all named authors to the manuscript.

Author Full Name:	Specification of Contribution to the Manuscript:
Jin Zhou, Limin Li	conception and design of the study, revise the manuscript
Jie Liu, Mengyuan Xu, Hongjuan Yu	designed the experiments
Chengfang Lv, Fenglin Cao, Zhenkun Wang	performed the experiments
Yueyue Fu, Mingwen Zhang, Hongbin Meng	sample selection
Xiaoqian Zhang, Liqing Kang, Zhuo Zhang, [illegible]	data analysis and interpretation
Jiawei Feng, Xin Lian, Lei Yu	drafted the manuscript

Please complete the table below to indicate the contributions of all named authors to the figures.

Figure 1:

Xiaoqian Zhang, Liqing Kang, Zhuo Zhang, Jinmei Li

Figure 2:

Xiaoqian Zhang, Liqing Kang, Zhuo Zhang, Jinmei Li

Figure 3:

Xiaoqian Zhang, Liqing Kang, Zhuo Zhang, Jinmei Li

Figure 4:

Xiaoqian Zhang, Liqing Kang, Zhuo Zhang, Jinmei Li

Figure 5:

Xiaoqian Zhang, Liqing Kang, Zhuo Zhang, Jinmei Li

Figure 6:

Xiaoqian Zhang, Liqing Kang, Zhuo Zhang, Jinmei Li

Signed for and on behalf of the Author(s):	Print Name:	Date:
Jin Zhou	Jin Zhou	2020-02-20
